# Expanding Diabetes Self-Management Education to Address Health-Related Social Needs: A Qualitative Feasibility Study

**DOI:** 10.3390/ijerph23010088

**Published:** 2026-01-08

**Authors:** Niko Verdecias-Pellum, Gianna D’Apolito, Abby M. Lohr, Aliria M. Rascón, Kelly N. B. Palmer

**Affiliations:** 1 College of Health Solutions, Arizona State University, Phoenix, AZ 85004, USA; gdapolit@asu.edu; 2 Department of Quantitative Health Sciences, Division of Epidemiology, Mayo Clinic, Rochester, MN 55905, USA; lohr.abby@mayo.edu; 3 Edson College of Nursing and Health Innovation, Arizona State University, Phoenix, AZ 85004, USA; amunoz8@asu.edu; 4 School of Medicine, University of Alabama at Birmingham, Birmingham, AL 35233, USA; kellypalmer@uabmc.edu

**Keywords:** health-related social needs, diabetes self-management education, community-based organizations, implementation science, social determinants of health

## Abstract

**Highlights:**

**Public Health Relevance—How does this work relate to a public health issue?**

- Diabetes self-management outcomes are strongly influenced by health-related social needs (HRSN), yet many diabetes self-management education (DSME) programs lack structured processes to identify and respond to these barriers, particularly in non-clinical settings.
- Community-based organizations deliver DSME to populations disproportionately affected by social and structural inequities, positioning them as critical but under-resourced sites for addressing HRSN within chronic disease management.

**Public Health Significance—Why is this work of significance to public health?**

- This study provides implementation-focused evidence on the feasibility of integrating HRSN screening into community-based DSME programs, addressing a critical gap between public health priorities and real-world delivery contexts.
- Findings highlight how misalignment between policy expectations, organizational capacity, and facilitator roles constrains equitable diabetes care, offering insight into why HRSN integration remains uneven despite growing emphasis in public health practice.

**Public health implications—What are the key implications or messages for practitioners, policy makers and/or researchers in public health?**

- Feasible integration of HRSN into DSME requires system-level supports including dedicated funding, clearly defined workforce roles (e.g., navigators or community health workers), and referral infrastructure rather than reliance on individual facilitators to absorb additional responsibilities.
- Policymakers and researchers should prioritize implementation strategies that align outer-setting expectations with inner-setting capacity in community-based settings, supporting early-stage feasibility and reducing unintended workforce burden.

**Abstract:**

Diabetes self-management education (DSME) programs are evidence-based interventions that improve glycemic control and self-care behaviors, yet their effectiveness may be limited by unaddressed health-related social needs (HRSN) (e.g., food insecurity, housing or utility instability, transportation barriers). This qualitative multiple case study examined the feasibility of integrating HRSN assessments into DSME delivery within three community-based organizations (CBOs) across urban and rural U.S. settings. Guided by the Consolidated Framework for Implementation Research, semi-structured interviews were conducted with 15 DSME facilitators and program leadership to identify contextual factors influencing implementation. Findings revealed that while DSME’s structured, manualized design promotes fidelity and client autonomy, it constrains responsiveness to the client’s HRSN. Facilitators expressed openness to integrating HRSN screening, particularly during intake, yet cited limited infrastructure, role clarity, and training as key barriers. CBOs were recognized as trusted, accessible spaces for holistic care, but growing expectations to address HRSN without adequate resources for referral created sustainability concerns. Participants recommended a parallel support model involving navigators or community health workers to manage HRSN screening and referrals alongside DSME sessions. Integrating HRSN assessment processes into DSME may enhance engagement, reduce attrition, and extend the reach of diabetes education to populations most affected by HRSN. However, successful implementation requires dedicated funding, workforce development, and cross-sector coordination. Findings underscore the importance of supporting CBOs as critical partners in bridging diabetes education and social care to advance whole-person, chronic disease management.

## 1. Introduction

Diabetes self-management is complex, especially among yet-to-be reached populations with limited resources. Effective diabetes self-management is critical in preventing complications, reducing healthcare expenditures, and enhancing quality of life among individuals living with diabetes [1,2,3,4]. Diabetes self-management education (DSME) is an evidence-based intervention that equips individuals with the knowledge and behavioral skills necessary to manage their condition [5,6]. While DSME has demonstrated efficacy in improving glycemic control, self-efficacy, and comorbidity prevention, its sustained effectiveness is inconsistently observed across all population groups [7,8,9]. A key factor limiting its impact is the presence of health-related social needs (HRSN), defined as individual-level, actionable and reportable conditions (e.g., food insecurity, inadequate transportation, housing instability) that may be identified through screening and addressed through linkage to services [7,8,9]. These HRSN arise from broader social determinants of health (i.e., upstream structural conditions) and can hinder engagement in DSME programs and recommended self-care behaviors [9,10].

Emerging evidence demonstrates that HRSN are linked to increased risk of diabetes and related comorbidities, higher prevalence and poorer management outcomes [9,10]. For example, lower socioeconomic status is associated with higher diabetes incidence, suboptimal glycemic control, and more severe complications [9]. Food insecurity can hinder adherence to DSME dietary recommendations, while transportation challenges and geographic isolation limit access to DSME programs, services, and essential supplies, especially among older adults [9]. Collectively, these social factors create compounding barriers that reduce DSME engagement and worsen diabetes outcomes [7,9,11]. Additionally, HRSN can impair decision-making and increase diabetes-related distress, further disrupting disease self-management [12,13,14]. Notably, studies have documented that 14–45% of individuals with chronic conditions experience at least one HRSN, and the prevalence of HRSN increases with the number of comorbidities [15,16,17].

Beyond their individual effects, HRSN often co-occur, creating a syndemic of deprivation that exacerbates the challenges of diabetes self-management [14]. Individuals facing multiple unmet needs must frequently make trade-offs between essential healthcare services (e.g., medications, appointments) and basic necessities (e.g., food, childcare, transportation), thereby undermining the effectiveness of DSME [18,19]. Despite robust evidence linking HRSN to diabetes outcomes, traditional DSME protocols continue to emphasize individual knowledge and behavior change, with limited integration of contextual assessments or tailored supports [7,20].

Emerging evidence highlights the importance of adapting DSME programs to more holistically address HRSN that shape patient behavior and health outcomes [21,22]. Recommendations include integrating validated HRSN screening tools into service workflows to inform individualized support and facilitate timely referrals [23]. Evidence suggests that doing so may enhance patient engagement in DSME, reduce distress, and improve clinical outcomes, especially in communities experiencing more HRSN [7,8,10,24]. The majority of literature has examined DSME from the perspective of clinical settings, leaving limited understanding of how these programs operate within community contexts [20,25,26,27]. Our study addresses this gap by providing insights from community-based organizations (CBOs). CBOs are uniquely positioned to serve populations experiencing higher levels of HRSN. However, they are often not included in clinical processes related to HRSN screening and documentation [7]. Consequently, key HRSN may remain unrecognized and unaddressed, undermining the reach and sustainability of DSME efforts [7,24].

While promising, efforts to integrate HRSN screening and referral processes into DSME remain underexplored in community-based DSME settings, where resource limitations, staffing constraints, and lack of implementation guidance can pose significant barriers. Furthermore, most existing DSME programs were developed prior to the healthcare system’s increased focus on addressing HRSN, and thus are not structurally equipped to respond to the multifaceted needs of their clients [9]. Even with strong empirical support for the efficacy of DSME, the lack of systematic strategies to assess and respond to HRSN represents a critical implementation gap.

In response, the present study examines the feasibility of integrating HRSN assessments alongside DSME protocols within CBOs using the Consolidated Framework for Implementation Research (CFIR) [28]. CFIR offers a comprehensive lens for exploring multi-level influences on implementation, including characteristics of the intervention, inner and outer organizational settings, individual implementers, and processes. Accordingly, this study focuses on feasibility as a pre-implementation outcome, examining whether and how HRSN screening could reasonably be integrated into CBO-led DSME programs and what organizational conditions would be required to support such integration. Drawing on semi-structured qualitative interviews with DSME facilitators and leadership across three US-based community organizations, this study identifies key barriers, facilitators and opportunities for embedding HRSN assessments and support services into DSME delivery. Findings contribute to an emerging body of literature on implementation strategies for whole-patient chronic disease management and inform future efforts to enhance DSME access, relevance and sustainability among hard-to-reach populations.

## 2. Materials and Methods

Study Design: We employed a qualitative multiple case study design grounded in implementation science. Guided by CFIR, the study explored the feasibility of integrating HRSN assessments within the standard DSME program delivery in CBO settings. Guided by implementation science, feasibility was conceptualized as the perceived appropriateness and operational fit of HRSN screening within existing DSME workflows, rather than as an evaluation of intervention (i.e., DSME) effectiveness or outcomes. This approach aligns with implementation-focused feasibility assessments conducted prior to intervention adaptation.

Setting: Data were collected from three CBOs delivering DSME programs, located in Appalachian West Virginia; St. Louis, Missouri; and Philadelphia, Pennsylvania. The West Virginia site is described at the regional level rather than by city because it encompasses a multi-county, predominantly rural area. Sites were purposively selected to capture variation across rural and urban contexts and diverse program structures. All CBOs offered DSME workshops on-site (e.g., at their facility, at a clinic, library, community center) and virtually throughout the region their respective organizations serve. Recruitment was conducted through a national social intervention network affiliated with clinics and CBOs who facilitate DSME programs. The PI contacted the network, which provided a list of potential CBO program sites. Program representatives were subsequently invited via email to participate in a one-time qualitative interview. The recruitment email outlined the study’s purpose. Four CBOs (two urban and two rural) were contacted, of which three agreed to participate and were scheduled for a virtual interview. One CBO did not respond to initial outreach nor two follow up email attempts. As a result, the participating sample may overrepresent CBOs with sufficient capacity or readiness to engage in research.

Sample: Semi-structured individual interviews were conducted with 15 staff engaged in DSME delivery (i.e., facilitators) and/or organizational leadership of participating CBO’s, including facilitators (*n* = 9), managers (*n* = 3), and individuals serving both roles (*n* = 3). Inclusion criteria included: (1) employment at a CBO providing DSME curriculum to adults with diabetes; (2) serving in a role that either delivers the DSME programming to clients or provides leadership oversight of DSME program delivery at the CBO; and (3) ability to speak and understand English. The research team did not collect additional demographic information to protect the confidentiality of participants. This sampling strategy was intended to capture perspectives from both front-line DSME facilitators and organizational leadership with a range of experience involved in program implementation.

Data Collection: Data collection occurred over a four-month period (April–August 2022). Each interview lasted a mean of 25 min (range: 22–35 min) and all interviews were conducted in English virtually via Zoom. The semi-structured interview guide consisted of 16 questions (see Table 1 for select questions). The Principal Investigator conducted and audio recorded all interviews after obtaining participant’s verbal consent. Recordings were then transcribed verbatim using a HIPAA-compliant transcription service (Landmark Associates, https://thelai.com/, URL accessed on 9 September 2025) [29]. The research team deleted recordings after completing transcription quality checks. Transcripts were de-identified prior to being uploaded into NVivo 14 (Version 14; Lumivero, Denver, CO, USA; 2023), a qualitative data analysis software [30].

Analysis: Although participants often used the terms social needs or social determinants of health interchangeably, these concepts were analytically distinguished in this study and operationalized as HRSN, a definition that was shared with participants at the onset of each interview. Participant terminology was retained in quoted excerpts to preserve meaning and context.

Data were analyzed using a two-phase, multi-step approach that combined both inductive and deductive strategies to enhance analytic rigor and interpretation [31]. In the first phase of analysis, we employed an inductive thematic approach to surface emergent themes across the data. Two members of the research team independently reviewed a subset of transcripts and conducted open coding, allowing for the identification of patterns and concepts grounded in participants’ narratives. Coders then met to compare their initial codes, reconcile discrepancies through structured team discussions, and collaboratively develop a focused coding framework reflecting the most salient and recurring topics. This refined framework captured core themes such as structural program design, participant characteristics and needs, delivery model adaptations, and perceptions of feasibility related to integrating HRSN assessments in complement to their DSME protocols.

In the second phase, a deductive framework analysis approach was informed by CFIR [31]. Building on the inductive codes and themes generated in Phase 1, the research team re-coded the data by mapping existing codes into CFIR’s predefined domains and constructs. These include Intervention Characteristics (e.g., adaptability, strength of evidence, perceived value), Inner Setting (e.g., organizational context where implementation occurs, including leadership support and readiness for change), Outer Setting (e.g., external influences such as client needs, community resources, policy requirements), Characteristics of Individuals (e.g., knowledge, self-efficacy, beliefs about the intervention), and Implementation Process (e.g., activities related to planning, engaging stakeholders, executing and evaluating). This approach enabled the refinement and organization of inductively derived themes within the CFIR framework, facilitating the interpretation of emergent insights through a theoretically grounded structure.

To enhance analytic rigor, a subset of transcripts was independently double-coded by two trained research team members. Coders met regularly to review CFIR-related code application and resolve discrepancies through structured team discussions focused on iteratively refining code definitions using a shared codebook. After the codebook was stabilized, remaining transcripts were single-coded with ongoing team discussion to ensure consistent application. Intercoder reliability was addressed through this interactive, team-based coding process rather than through calculation of formal agreement statistics.

Thematic saturation was assessed iteratively and was reached when no new themes emerged across later interviews. The final sample of 15 interviews across the three CBOs was sufficient to achieve thematic saturation and robust coverage of key CFIR constructs. The research team maintained an audit trail documenting analytic decisions and periodically convened to review summaries, interpret patterns, and triangulate findings across the study sites. This iterative and collaborative process supported the identification of cross-cutting themes and site-specific nuances, contributing to a comprehensive understanding of implementation barriers and facilitators relevant to integrating HRSN screening into community-based DSME programs.

The study protocol was initially reviewed by the IRB at Washington University in St. Louis and granted exempt status, with a waiver of written consent. Following the principal investigator’s institutional transition, the protocol was transferred and granted exempt status by the Arizona State University IRB. Although the study qualified for exemption as minimal risk, organizational-level qualitative research and did not involve the collection of personally identifiable information, all participants provided verbal informed consent prior to participation, including consent for audio recording. This approach aligned with IRB guidance for ethical conduct of exempt qualitative research.

## 3. Results

Overall, participants had a mean of 9.8 years (range: 2–27 years) of experience in their current roles. Among DSME facilitators, training backgrounds included completion of an accredited DSME certification program (*n* = 4) (e.g., American Diabetes Association recognized program) or a structured workshop facilitator training program (*n* = 8) (e.g., Stanford Model, Diabetes Prevention Program).

Drawing on participant perspectives, we present themes using the CFIR framework to explore contextual determinants influencing the feasibility of integrating HRSN screening and services alongside DSME programs delivered by CBOs. Our findings were largely consistent across urban and rural settings, with differences primarily reflecting variation in resource availability and community referral density rather than divergence in core implementation challenges. Ten core themes were organized under the five CFIR domains. Table 2 summarizes the key findings in the context of the CFIR framework.

### 3.1. DOMAIN 1: Intervention Characteristics

Participants were asked about the structure and design of the DSME programs they deliver. They shared perspectives on the benefits and challenges of standardized delivery models, including how program fidelity promotes consistency and supports client autonomy, but may also limit responsiveness to clients’ individual and contextual needs such as HRSN. The results in Themes 1 and 2 reflect these views.

#### 3.1.1. Theme 1: Strengths and Boundaries of DSME Structure

With DSME representing the evidence-based intervention under study, rather than HRSN screening, it was first necessary to understand the DSME program structure itself. Participants consistently characterized DSME protocols as highly structured and manualized, highlighting both consistency and constraints. These six-week, group-based workshops, delivered by trained lay leaders, were described as centering on scripted facilitation, peer learning, and client-driven action planning. The standardized curriculum was viewed as both a strength that promotes autonomy and reproducibility and a limitation insofar as its rigidity constrained facilitators’ ability to adapt content to clients’ evolving needs.

##### Perceived Strengths of Structure and DSME Client Autonomy

As depicted by the following quotes, several participants emphasized that the structured design of DSME programs supported client engagement and self-directed behavior change by providing clear guidance while preserving participant autonomy.

*“So I will say that I think the action planning, the fact that it is so structured, is great. I think anybody can make use of having a structured way to think through a problem or an action.”*

—CBO Manager, W. Virginia.

*“There’s guidance on how to develop an action plan, but the action plan that the client chooses is of their own. They choose it. It provides an authenticity to the program.”*

—DSME Facilitator, St. Louis.

##### Perceived Limitations and Challenges to Responsiveness

In contrast, facilitators also described limited adaptability within the DSME curriculum, noting that strict fidelity requirements constrained their ability to respond to emergent HRSN or integrate local resource information. This conflict between fidelity and adaptability was consistently described across sites.

*“We always have inquiries for needed resources that come up within our DSME program. We’re not given a list of resources. Sometimes we provide some, but it’s very inconsistent.”*

—DSME Facilitator, Philadelphia.

*“We’re not allowed to stray from the script. Even if someone brings up something outside of the curriculum, I try to redirect or follow up after class.”*

—DSME Facilitator, St. Louis.

#### 3.1.2. Theme 2: Fidelity Requirements and Adaptation Constraints

Participants consistently framed fidelity as both a safeguard for program integrity and a barrier to innovation, highlighting how concerns about deviating from evidence-based protocols limited openness to integrating HRSN screening directly into DSME sessions. While DSME facilitators acknowledged the importance of maintaining evidence-based delivery, they also expressed a desire for greater flexibility and training to better address complex client needs, as exemplified by these quotes.

*“If you start integrating other stuff, even something like a social needs screener, you’re not really doing the program the way it was designed. I’d be worried about getting off track. We’re trained to stick to the content as-is to preserve fidelity.”*

—DSME Facilitator, W. Virginia.

*“We have a structured class. We’re not really supposed to give advice. That’s the facilitator’s role—just to keep it to the manual. However, it could be helpful to have the opportunity to talk about client’s other needs and to be trained on how to help them.”*

—DSME Facilitator, W. Virginia.

### 3.2. DOMAIN 2: Inner Setting

When asked whether and how HRSN emerge during DSME, and at what points screening might be integrated into the program, participants described both formal opportunities and informal instances when HRSN surfaced organically. Themes 3 and 4 reflect these perspectives.

#### 3.2.1. Theme 3: Entry Points for HRSN Screening

**Figure 1:**
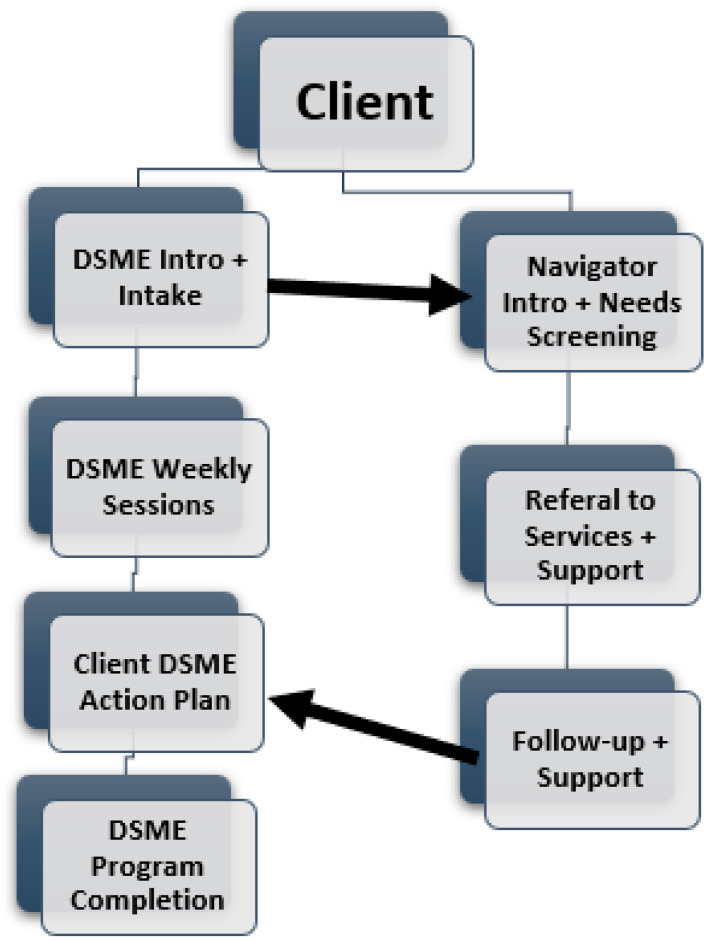
Conceptual Model for Integrating Health-Related Social Needs into DSME Programs.

Within the inner setting domain, participants identified intake and early engagement as compatible and operationally feasible points for HRSN screening, describing enrollment as a natural moment to introduce brief screening tools without disrupting DSME curriculum flow or group cohesion. Figure 1 presents a conceptual model derived from recurring, cross-site patterns identified during analysis of participants’ descriptions of feasible workflows for integrating HRSN screening into DSME programs, including intake-based screening and role differentiation between DSME facilitators and other client support staff.

*“We already have them fill out a form the first day, checking their chronic conditions, go over goals and medication lists. Adding something about needs wouldn’t be hard logistically.”*

—CBO Manager, W. Virginia.

*“Now, frequently when we get started… usually that first week or so… we give each one a call, just saying again who we are, ‘How’s everything going? Do you have any questions…? This would be a good time to ask about social needs also.”*

—CBO Manager, Philadelphia.

#### 3.2.2. Theme 4: Informal Emergence of HRSN

In addition to formal intake opportunities, facilitators described HRSN disclosures as emerging informally during unstructured moments such as breaks, post-session conversations, or peer discussions. This underscores the absence of standardized inner setting processes to systematically capture or respond to these needs despite their frequent relevance to participation.

*“People share a lot after class. That’s when you find out they’re having transportation issues or can’t afford groceries. You could tell they were more anxious and stressed… those were the ones that were asking more questions.”*

—DSME Facilitator, St. Louis.

*“There’s no formal way we do this... it’s more within classes when people raise these things [social needs]. We’re not supposed to respond, even if we know the answer. We help them phrase their concern and encourage them to call their provider.”*

—DSME Facilitator, W. Virginia.

### 3.3. DOMAIN 3: Outer Setting

Participants were asked about the external factors influencing CBOs’ ability to support DSME program clients with HRSN. Theme 5 captures perspectives on shifting expectations and system-level constraints.

#### Theme 5: Policy Expectations and Capacity Misalignment

Participants articulated a growing expectation for CBOs to address HRSN, reflecting outer setting pressures related to evolving healthcare norms and increased emphasis on social care integration. These expectations were described as emerging despite the absence of formal mandates specific to lay-led DSME programs and externally driven relative to available organizational capacity.

*“There’s definitely pressure to do more around social needs. Even if it’s not required yet, it feels like it’s coming.”*

—CBO Manager, St. Louis.

*“I think the expectation is shifting—community orgs are being asked to pick up what health systems don’t catch.”*

—CBO Manager, Philadelphia.

Participants emphasized that limited reimbursement pathways and billing constraints restricted CBOs’ ability to formalize and operationalize HRSN-related activities, even when staff expertise or partnerships existed. In the absence of reimbursement mechanisms, policy guidance, and formal referral infrastructure, participants described challenges integrating HRSN screening and follow-up into DSME delivery.

*“We’re being asked to do more, even if we’re not reimbursed. It’s like there’s this push to be part of the healthcare net without the resources.”*

—CBO Manager, St. Louis.

*“We do have people that have that kind of outreach experience that could be activated… But the issue usually with health centers is if there’s money for something, they’ll do it. To be billable, you had to have someone who met the criteria for Medicare rules. And [large healthcare system] did not want to be a billing partner... they just never were able to get the logistics.”*

—CBO Manager, Philadelphia.

Participants also highlighted unclear role boundaries as an external constraint, noting that expectations to address HRSN increasingly extended into activities typically associated with clinical or case-management roles.

*“We can’t be expected to take on clinical roles. We’re not trained for that, and we don’t have the infrastructure.”*

—CBO Manager, St. Louis.

*“We’re trained to facilitate—not handle referrals or social services. If something serious comes up, we pass it on.”*

—DSME Facilitator, Philadelphia.

Participants linked outer setting social conditions such as housing instability, food insecurity, and transportation barriers to challenges in DSME attendance and engagement, describing how these contextual factors shaped who was able to participate consistently.

*“It implies a level of stability to even make it to the weekly class… if you’re dealing with housing issues, you might not be showing up.”*

—DSME Manager & Facilitator, W. Virginia.

### 3.4. DOMAIN 4: Characteristics of Individuals

Analysis within this domain focused on DSME facilitators and program managers involved in implementation, rather than DSME clients. Participants were asked to reflect on their roles in addressing HRSN, the challenges encountered during DSME delivery when such needs arise, and perceived barriers to integrating HRSN-related supports into their programs. Theme 6 reflects these perspectives.

#### Theme 6: Emotional Burden and Uncertainty in Responding to Serious Needs

Within the characteristics of individuals domain, facilitators’ accounts reflected role ambiguity and reduced self-efficacy when encountering serious social needs, contributing to reluctance to initiate HRSN-related conversations in the absence of clear guidance or support.

*“If someone’s desperate, like housing or safety issues, I wouldn’t know what to do beyond listening. There are so many different levels of need. Some come to us because they’re at a loss, with no support. “I don’t know, but I’ll see what I can find for them if it’s something... within our scope of practice.”*

—DSME Manager & Facilitator, St. Louis.

*“We don’t respond, even if we know the answer... we make that referral for them to talk to their healthcare provider.”*

—DSME Facilitator, W. Virginia.

Several DSME facilitators described emotional strain associated with hearing about clients’ challenges without having the tools or authority to meaningfully intervene. This was accompanied by reluctance to initiate conversations about HRSN, particularly when facilitators were unsure whether appropriate follow-up or support could be provided.

*“It doesn’t feel right to ask those questions if we can’t do anything to help. That’s just opening wounds.”*

—DSME Facilitator, Philadelphia.

*“We’re not caseworkers. It’s an emotional burden to hear things you can’t help with.”*

—DSME Facilitator, W. Virginia.

### 3.5. DOMAIN 5: Process

Participants were asked how HRSN-related needs emerge during the DSME delivery and the processes that might strengthen integration of HRSN-related services. Participants discussed how implementation processes, workflows, and coordination shaped client engagement and program outcomes. Themes 7–10 their perspectives.

#### 3.5.1. Theme 7: HRSN as Hidden Drivers of DSME Attrition and Disengagement

Participants described client attrition and disengagement as closely tied to unaddressed HRSN, reflecting CFIR process challenges related to the absence of systematic mechanisms for identifying, documenting, and responding to social barriers.

*“Without asking, we miss the real reasons people aren’t engaging or coming back.”*

—DSME Facilitator, Philadelphia.

*“If you’re really struggling—housing, transportation—you may not even be in the class. That’s who we’re missing.”*

—DSME Facilitator, W. Virginia.

*“They stop coming, and we don’t know why. You start to realize it’s more than just interest—it’s survival.”*

—DSME Facilitator, St. Louis.

Participants also noted how the absence of structured HRSN identification shaped program reach, with continued participation more common among individuals with greater stability rather than those facing higher levels of unmet need.

*“If we don’t find out what’s getting in their way, we’re only reaching the people who already have what they need. We’re only seeing the people who manage to show up. Those with the greatest needs may never get in the room.”*

—DSME Facilitator, Philadelphia.

*“The people who drop out are usually the ones with the biggest challenges—housing, food, safety. If we don’t ask, we lose them. If you don’t address these barriers, it’s not really self-management. It’s survival management”*

—DSME Facilitator, St. Louis.

Participants further described concrete material constraints affecting clients’ ability to engage in DSME and implement self-management recommendations, including limited access to affordable food options.

*“We’re expecting them to pay for fruits and vegetables… the only thing that’s even close is a dollar general or a family dollar which is all very much processed foods.”*

—DSME Facilitator, Philadelphia.

#### 3.5.2. Theme 8: Valuing CBOs as Trusted Settings for Engagement and Referrals

Participants emphasized CBO-based DSME programs as trusted and accessible implementation contexts, reflecting CFIR process and inner setting conditions that influenced trust and willingness to disclose HRSN.

*“Churches, libraries, senior centers—we’re in places where people already feel safe and supported. That helps with trust.”*

—DSME Facilitator, Philadelphia.

*“We go into churches, senior centers, libraries… being in a community location is more comforting than a hospital setting. People are more willing to open up when you’re in their community.”*

—DSME Facilitator, St. Louis.

Participants described identifying HRSN and initiating referrals through informal, relationship-based processes during DSME delivery. In several cases, DSME programs were co-located with or directly connected to other community services, including food assistance, housing support, and mental health resources, which participants noted facilitated referral and follow-up activities. These relational and logistical connections were described as occurring alongside DSME delivery, even when they were not formally embedded within DSME workflows.

*“There’s a lot of different community partners... So we can be that organization that can really connect people outside of the program to these other things.”*

—CBO Manager, St. Louis.

*“We work with... [social services partner]... for all types of resources from transportation to food assistance, housing.”*

—CBO Manager, W. Virginia.

Participants noted the credibility of host organizations, particularly those affiliated with large healthcare or nonprofit systems, as influencing client trust and engagement in DSME programs. Facilitators noted that clients were more likely to engage and disclose social challenges when programs were perceived as reputable and connected to established systems. Participants also described how trusted organizational affiliations, community ties, and access to broader support services shaped referral opportunities and client perceptions of program legitimacy.

*“We have [hospital name] partners... we have access to these types of people that we can make a stronger connection with that could potentially be a referral source.”*

—CBO Manager, W. Virginia.

*“Being a part of the [hospital name] system is a good thing. If the hospital’s supporting the program, even if unofficially, that means the program is a good program and it can be trusted.”*

—CBO Manager, St. Louis.

#### 3.5.3. Theme 9: Recommendations for Parallel Support Structures

Participants recommended navigator- or care coordinator-led models as process-level implementation strategies to support HRSN screening and follow-up alongside DSME delivery while preserving curriculum fidelity, consistent with the workflow model illustrated in Figure 1.

*“It makes more sense to have a separate person for that—a navigator or care coordinator—so we don’t disrupt the classes. Someone outside the group who can follow up and make those connections would be best.”*

—DSME Facilitator, W. Virginia.

*“We already have clinic partners and collect info at the start. So a navigator role could use that info to follow up.”*

—DSME Manager, St. Louis.

Participants expressed how these roles could collaborate with healthcare systems and leverage existing partnerships to support HRSN-related follow-up activities alongside DSME delivery. These functions were described as occurring in parallel to DSME facilitation rather than within core educational sessions.

*“It would be more like a patient navigator… someone that worked with the healthcare system… who would do the follow-up.”*

—DSME Facilitator, St. Louis.

Participants described existing partnerships and infrastructure as shaping how HRSN-related referrals occurred within DSME programs. Across sites, participants highlighted relationships with local food banks, area agencies on aging, clinics, and faith-based organizations, noting that these connections supported informal referral practices. These partnerships were described as operating alongside DSME delivery and varied in formality across programs.

*“It works better when the community comes together—different agencies playing their part. We have partners we could refer to—we just need a formal process.”*

—DSME Manager, St. Louis.

*“There’s always someone at the facility—a parish nurse, a center coordinator—who knows the local services better than we do. So I’m a big fan of any kind of outreach or any kind of navigation… [leaders] are more aware of how to speak to others… in a supportive role.”*

—DSME Manager, W. Virginia.

*“We get clinic referrals, run programs in community centers, and partner churches—there’s a lot already in place.”*

—DSME Manager, St. Louis.

Participants shared possible ways that additional HRSN-related support roles could influence who enrolls in and remains engaged in DSME programs, particularly among individuals who currently seek services elsewhere for social support.

*“If you had somebody who could help clients with that... that might draw more people to the program… Right now I’m not getting those people because they’re going elsewhere for those [social] needs.”*

—CBO Manager, Philadelphia.

*“That would be my dream... someone who acts as their coordinator to help them get access to the systems they need.”*

—CBO Manager, St. Louis.

#### 3.5.4. Theme 10: What Keeps Participants Engaged

Participants described continued engagement in DSME programs as influenced by both educational content and the extent to which programs acknowledged and responded to clients’ social context, noting that ongoing support and attention to real-life circumstances shaped whether participants remained engaged.

*“People want these programs to continue. The support is part of what keeps them going—not just the content.”*

—DSME Facilitator, St. Louis.

## 4. Discussion

This study examined the contextual factors influencing the feasibility of integrating HRSN screening and services alongside DSME programs delivered by CBOs, using CFIR as an organizing lens. Findings illustrate how feasibility is shaped by interacting influences across intervention characteristics, inner and outer settings, characteristics of individuals delivering DSME, and implementation processes. Importantly, intervention characteristics and inner setting domains were more analytically developed due to participants’ direct experience with existing DSME programs, whereas process and outer setting domains reflected more emergent and less formalized conditions. This pattern is consistent with an early-stage feasibility assessment, in which implementation processes and system-level supports have not yet been fully operationalized.

### 4.1. Outer-Setting Pressures and Structural Constraints on Feasibility

A central finding relates to the misalignment between growing expectations for CBOs to address HRSN and the absence of corresponding policy mandates, reimbursement mechanisms, or referral infrastructure to support such efforts. Participants described increasing pressure to respond to HRSN despite limited institutional resources, framing feasibility as a structural rather than motivational challenge. Even when staff expertise or community partnerships existed, the lack of sustainable funding pathways and formalized workflows constrained organizations’ ability to operationalize HRSN screening and follow-up. These findings underscore how outer-setting pressures can outpace organizational capacity, placing CBOs in a position of responding to HRSN without the system-level supports typically required for sustained implementation. These outer-setting pressures interact with inner-setting constraints such as limited staffing, informal workflows, and curriculum rigidity. They also manifest at the individual level as increased facilitator burden and reluctance to initiate HRSN-related conversations. These challenges point to gaps in role clarity, emotional support, and organizational readiness, echoing prior research on the burdens faced by lay workers navigating structural complexity without adequate tools [32,33].

Participants also emphasized that broader social conditions such as housing instability, food insecurity, and transportation barriers directly shape DSME attendance and engagement. These outer-setting factors not only influence whether individuals enroll in DSME programs, but also whether they are able to participate consistently. Together, these findings highlight how feasibility cannot be disentangled from the structural and material contexts in which DSME is delivered.

### 4.2. Process Gaps, Attrition, and the Limits of Education-Only Models

Process-related findings further illuminate how the absence of systematic HRSN identification and response mechanisms contributes to disengagement and attrition. Facilitators reflected on missed opportunities to identify barriers early, noting that health behavior change cannot occur in isolation from the structural conditions that shape individuals’ daily lives. Without formal screening or follow-up processes, attrition often occurred without clear explanation, affecting individuals with higher levels of unmet HRSN. These patterns reflect CFIR process challenges related to the absence of formal mechanisms for identification, follow-up, and adaptive response.

These findings challenge education-only models of chronic disease management and reinforce the need for implementation strategies that acknowledge and address the social context of self-management. Feasibility, in this sense, extends beyond whether HRSN screening can be added to DSME workflows to whether programs are equipped to respond meaningfully once HRSN are identified.

### 4.3. Trusted Community Settings as Enablers of Engagement and Disclosure

Despite these challenges, participants consistently identified CBO-based DSME programs as trusted and accessible platforms for engagement. Delivering DSME in familiar, community-based spaces such as churches, libraries, and senior centers was described as fostering relational safety and trust, increasing the likelihood that clients would disclose HRSN. These non-clinical environments were perceived as more welcoming and less stigmatizing than traditional healthcare settings, enabling more open conversations about HRSN challenges.

Participants also described how co-location with, or proximity to, other community services facilitated informal referrals and follow-up activities. While these processes were often relationship-based and not formally embedded within DSME workflows, they played a critical role in connecting clients to needed resources. These findings highlight how trust, organizational credibility, and community ties function as important process-level facilitators of HRSN integration, even in the absence of formal infrastructure. Notably, prior studies have demonstrated that DSME programs delivered in trusted settings and suggest that brief, intake-based HRSN screening can facilitate early identification of needs without disrupting program delivery, suggesting that process gaps observed here are not inherent to DSME but reflect implementation design choices [20,24,34,35,36,37,38].

### 4.4. Parallel Support Structures as a Feasible Implementation Strategy

To reconcile growing expectations with limited DSME facilitator capacity, participants emphasized the need for parallel support structures such as care coordinators, patient navigators, or community health workers to operate alongside DSME programs. Rather than embedding HRSN screening and referral responsibilities within DSME facilitation itself, these roles were viewed as a way to preserve curriculum fidelity while extending program capacity to address HRSN. As illustrated in Figure 1, such models reflect DSME facilitator-identified strategies for distributing responsibility across roles and creating clearer boundaries between education and social care functions, and are intended to illustrate feasible implementation pathways rather than represent a tested or prescriptive model.

These parallel roles were described as particularly well-suited to leveraging existing partnerships with healthcare systems and community organizations, enabling coordinated follow-up and strengthening cross-sector linkages between clinical and community settings. In doing so, participants described DSME as a more holistic platform for advancing health and sustained behavior change [39]. Importantly, these approaches were framed as feasible adaptations within current resource constraints, rather than as fully realized implementation models.

### 4.5. Engagement as a Function of Social Responsiveness

Finally, participants reflected on what sustains engagement in DSME programs, emphasizing that continued participation is shaped not only by educational content but also by whether programs acknowledge and respond to clients’ social realities. Support, empathy, and responsiveness to real-life circumstances were described as critical to sustaining engagement over time. Without attention to HRSN, clients may be left managing health behaviors within environments that constrain their ability to act, limiting the impact of DSME programs. This aligns with CFIR characteristics of individuals and process domains, highlighting how facilitator capacity and program responsiveness shape sustained engagement.

### 4.6. Implications for Feasibility and Future Implementation

These findings are derived from a small number of well-established, relatively well-resourced CBOs with mature DSME programs, and feasibility may differ in smaller, newer, or less-resourced community organizations. Taken together, these findings suggest that feasibility of HRSN integration within CBO-led DSME programs depends on aligning outer-setting expectations with inner-setting capacity and process-level supports. While CBOs are well positioned to engage clients and identify HRSN, sustainable integration will require policy-level investment, reimbursement mechanisms, and workforce models that extend beyond education alone. Strengthening formal partnerships with organizations that address food, housing, and other HRSN can streamline referrals and foster shared accountability [40,41,42]. Future implementation efforts should prioritize scalable, role-differentiated approaches that leverage trust-based community settings while addressing structural barriers to participation and engagement.

This study has several limitations. First, to protect participant confidentiality and organizational anonymity, demographic characteristics (e.g., gender, age, race/ethnicity) was not collected. While this approach minimized the risk of deductive disclosure given the small size of DSME facilitator teams at participating sites, it limits the ability to assess how perspectives may differ across participant identities. However, their range of tenure (2–27 years), enhances insight into implementation across experience levels. Second, the interviews were conducted virtually via Zoom, which enabled participation across geographically diverse sites but limited direct observation of non-verbal cues and in-person contextual dynamics. Third, our findings are based on three well-established CBOs with mature DSME programs and may not generalize to newer or less-resourced, or international settings, where DSME infrastructure, workforce capacity, and referral networks may be more limited. In such contexts, feasibility may depend on leveraging informal or non-traditional partnerships (e.g., faith-based organizations, local NGOs, or community volunteers), task-shifting HRSN screening and follow-up to existing community or lay health worker models, and integrating brief, low burden, low cost (e.g., paper-based) screening tools into routine intake or outreach activities rather than formal program workflows. These adaptations underscore the importance of contextual flexibility and local capacity building when translating HRSN integration strategies across diverse resource settings. Fourth, the perspectives captured reflect those of program staff and leadership, not clients. The absence of client perspectives limits insight into how individuals with diabetes perceive, prioritize or experience HRSN screening within DSME programs. Future research should incorporate client voices to assess the acceptability, perceived burden, and relevance of incorporating HRSN screening and referral into DSME programs. Additionally, participants’ professional roles and strong commitment to DSME programs may have contributed to social desirability bias, potentially shaping how challenges, limitations, or areas of concern were described. Finally, findings reflect a snapshot in time during a rapidly evolving policy environment around HRSN and community health. As such, the study provides timely insight into implementation considerations and organizational decision-making during a period of transition, when formal guidance and infrastructure are still emerging. These findings establish a valuable baseline for understanding early-stage feasibility as policy and reimbursement structures continue to develop.

## 5. Conclusions

The DSME model offers a promising, contextually grounded platform for advancing upstream approaches to care, particularly due to its foundation in community trust and accessibility. Delivering DSME in safe, non-clinical settings may enhance engagement and create an opportunity to systematically identify and address barriers associated with HRSN. Without formal mechanisms to identify HRSN, these programs risk excluding those most affected by limited resources. Thoughtful, resource-informed integration of HRSN--supported by appropriate funding, staffing, and infrastructure--can empower DSME programs to serve as a critical bridge between health education and the social factors that shape its effectiveness. CBOs, often key access points for yet to be reached populations, must be equipped with tools, training, partnerships, and policies that support HRSN screening and referrals without overburdening staff. Future efforts should prioritize cross-sector collaboration, community health worker or navigator-led models, and trauma-informed training to ensure HRSN are met and DSME remains a sustainable model of whole-person care.

## Figures and Tables

**Table 1:** Excerpt of Key Questions from the Interview Guide.

How is the diabetes self-management education (DSME) material delivered? For example, are they group workshops or one or more 1-on-1 sessions? [If a workshop] How many clients are in a typical workshop cohort? [If a workshop] Are there any 1-on-1 components between staff and the clients during the workshop series? If so, what topics are covered at that time? [If one-on-one] What topics are covered during the 1-on-1 sessions? [If one-on-one] How many times do staff meet with a client? Is an action plan developed? What does that entail? What are some challenges staff have in engaging clients to receive DSME? - Probes: What are some challenges staff have observed regarding client’s ability to reach the goals they plan for their DSM? - What are the reasons clients might refuse to receive DSME? - What are the reasons clients might be ineligibility to receive DSME? What are some factors or resources that staff find helpful in engaging clients to receive DSME? What about tools that help clients to reach their DSM health goals? Interviewer Script: Next are some questions about health-related social needs (HRSN) your clients may have. HRSN include food insecurity, housing instability, financial hardship including employment, and challenges with transportation, child care or safety. Do clients go through a routine assessment of their HRSN as part of their care plan? What about during DSM education? [If yes to either] Please tell me about the process for doing this and how that information is used as part of their care and services. Do clients talk about HRSN in relation to their DSM during the sessions? What types of social needs come up in these discussions? Probe: In addition to the HRSN you mentioned, do staff address (name the ones not mentioned)? a1. Housing instability YES__ NO __ a2. Food insecurity YES__ NO __ a3. Food deserts YES__ NO __ a4. Transportation barriers YES__ NO __ a5. Childcare barriers (e.g., access, cost) YES__ NO __ a6. Neighborhood safety YES__ NO __ a7. Personal safety YES__ NO __ a8. Financial hardship (e.g., Not enough money for utilities, necessities; not enough for unexpected expenses; unemployed/low-income/poverty) YES__ NO __ [If yes to any] What have clients shared about their concerns/experiences with this/these HRSN(s) [name the ones noted above]? Please describe if there are particular moments/topics in the DSME that HRSN tends to come up in discussion. For example, when staff are talking about nutrition and a client mentions not having access to a grocery store or not having enough money to buy healthier food options. Do staff have any approaches that enable them to help clients address HRSN? If not, what do you think might be helpful? If [name organization] were to integrate HRSN navigation services for clients managing diabetes, what existing internal resources do you think would be most useful to develop that service? Probe: What does [name organization] already have in place that can strengthen action to add HRSN navigation? What additional non-existing internal resources do you think would be needed? What are [name organization]’s existing external resources (e.g., partners, community ties) that you think would be most useful to connect your clients to HRSN services? What additional non-existing external resources do you think would be needed? Which staff roles would be best suited to identify and facilitate HRSN navigation services at [name organization]? What, if any, additional training do you think would be needed for that staff? Do you think an additional dedicated staff member would need to be hired for this service? What challenges would you anticipate in adding HRSN navigation within [name organization]? What challenges to clients would you anticipate?

**Table 2:** Key findings in the context of the CFIR Framework.

CFIR Domain	Construct	Key Findings
Intervention Characteristics	Relative advantage, design quality	DSME’s structured, evidence-based design promotes participant autonomy and fidelity, but limits responsiveness to evolving HRSN and contextual realities.
	Adaptability	Integrating HRSN screening directly into core sessions is challenging; more feasible during intake or via parallel workflows. Flexibility varies across sites and roles.
Inner Setting	Compatibility, available resources	HRSN screening aligns with DSME mission and can fit naturally into intake or post-session engagement. However, staffing and infrastructure are insufficient for follow-up.
	Implementation climate	There is openness to innovation, but integration requires additional funding, clearly defined roles, trauma-informed training, and leadership support.
Outer Setting	External policy, incentives	CBOs anticipate increasing pressure to screen for HRSN, despite limited reimbursement, inconsistent infrastructure, and lack of federal/state mandates specific to DSME.
	Community linkages, expectations	Partnerships with clinics, churches, and community agencies exist and support informal referrals; however, formal coordination mechanisms are underdeveloped, limiting organizational capacity.
Characteristics of Individuals	Self-efficacy, role clarity, and alignment with CBO	Facilitators are committed and trusted by participants but feel ethically conflicted and underprepared to address serious HRSN without adequate training and support.
Process	Planning, engaging, delivery	Facilitators widely endorse a navigator or care coordination model to manage HRSN screening and referrals outside of DSME sessions. Referral and tracking systems remain limited.
	Reflection	Unaddressed HRSN and limited screening processes were described as contributing to participant attrition, with continued participation more common among individuals with greater stability.

## Data Availability

The original contributions presented in this study are included in the article. Further inquiries can be directed to the corresponding author.

## Abbreviations

The following abbreviations are used in this manuscript:

DSME	Diabetes self-management education
HRSN	Health-related social needs
CBOs	Community-Based Organizations
CFIR	Consolidated Framework for Implementation Research
